# Major depressive disorder in detention officers

**DOI:** 10.11606/s1518-8787.2021055002507

**Published:** 2021-04-05

**Authors:** Sheila Nascimento Santos, Kionna Oliveira Bernardes Santos, Fernando Martins Carvalho, Rita de Cássia Pereira Fernandes

**Affiliations:** I Universidade Federal da Bahia Programa de Pós-Graduação em Saúde, Ambiente e Trabalho Departamento de Medicina Preventiva e Social SalvadorBahia Brasil Universidade Federal da Bahia. Programa de Pós-Graduação em Saúde, Ambiente e Trabalho. Departamento de Medicina Preventiva e Social. Salvador, Bahia, Brasil

**Keywords:** Prisons, Depressive Disorder, Major, Patient Health Questionnaire, Occupational Health

## Abstract

**OBJECTIVE:**

To identify factors associated with major depressive disorder (MDD) in detention officers.

**METHODS:**

This cross-sectional study included all detention officers from the largest prison complex in the state of Bahia, Brazil. A self-reported questionnaire collected sociodemographic, occupational and health data. The outcome variable – MDD – was evaluated by the Patient Health Questionnaire-9 (PHQ-9) and classified by the cut-off point ≥ 10 method and the algorithm method. The association measure used was the prevalence ratio (PR). Following Cox multivariate regression, the variables were divided into two blocks: sociodemographic characteristics and work, in that order. Only variables with adjusted PR (PR_adj_) ≥ 1.30 were selected to compose the final models.

**RESULTS:**

The MDD prevalence by the cut-off point ≥ 10 (simple) and algorithm method in the 401 officers investigated was 18.8% and 9.3%, respectively. MDD prevalence by cut-off point ≥ 10 was higher in female officers (PR_adj_ = 2.77), who suffered threat from factions (PR_adj_ = 2.05), did not report institutional training for the position (PR_adj_ = 1.38), stated that the environment and working conditions interfered in their physical health (PR_adj_ = 3.51) and performed stress-generating activities (PR_adj_ in increasing gradient). MDD prevalence by the algorithm method was higher in female agents (PR_adj_ = 3.45), with tertiary education (PR_adj_ = 1.71), who stated that the environment and working conditions interfered in their physical health (PR_adj_ = 6.33), suffered threat from factions (PR_adj_ = 2.14), did not report institutional training (PR_adj_ = 1.50) and have frequent contact with inmates at work (PR_adj_ = 1.48).

**CONCLUSION:**

The high MDD prevalence in these detention officers was associated with sociodemographic factors and, especially, aspects of their work.

## INTRODUCTION

Prison work is a stressful, risky occupation stigmatized by society^1,2^. The detention officer (AP) is responsible for the internal security of prisons and individuals deprived of their liberty^3^. Among other tasks, these professionals are charged, daily and continuously, with searching inmates, cells and visitors; opening and closing prison cells; performing internal surveillance of units; managing inmates inside and outside the prison; and disciplining their meals^4^.

AP’s work is characterized by direct contact with inmates, specific service regulations, environments with poor infrastructure, profession without legal recognition as a public security function, pressure, work overload, devaluation and lack of social recognition of the importance of their craft. APs are exposed to high psychosocial risks arising from their work, making them prone to common mental disorders, psychotic disorders, chemical dependence, alcoholism, stress, anxiety, burnout, work leave and depressive syndromes^3,5,6^.

Depressive disorders stand out among mental disorders due to their high prevalence in the population, associated with suffering or impairment, which can affect social and professional activities^7^. Given this context, this study aimed to identify factors associated with major depressive disorder (MDD) in AP.

## METHODS

This is a cross-sectional epidemiological study. Its population comprised all AP, without gender or age restriction, from the effective staff of the Secretariat of Prison Administration and Resocialization (Seap) of Bahia, distributed among leadership, administration and security positions and based in the Mata Escura Prison Complex, in the Metropolitan Region of Salvador (Bahia, Brazil). Data collection took place from August to December 2018.

The Prison Complex comprises eight units with their own characteristics related to the inmate’s legal status and the nature of each activity performed there: (1) Prison Monitoring Center (COP), with 96 vacancies, where its conducted general and criminological screenings of convicted inmates, as well as special, temporary, or convicted inmates, over 60 years of age, and other tests authorized by the State Inspector General’s Office; (2) Prison Medical Center (CMP), with 16 vacancies, which provides emergency medical care to inmates of both genders, from the various Prison Units in the state of Bahia; (3) Special Group for Prison Operations (GEOP), exclusively intended for deploying specialized prison intelligence intervention agents; (4) Female Prison Unit (CPF), with 132 vacancies for the custody of convicts in closed and semi-open regimes from countryside counties and provisional detainees from the county of Salvador – almost all AP of this unit are women; (5) Salvador Public Prison (CP), with 1,092 vacancies, which holds provisional detainees from the capital and, exceptionally, from the countryside, when authorized by the State Inspector General’s Office; (6) Prison of Salvador (PS), with 784 vacancies, which houses provisional detainees, similar to those found in CP; (7) Special Disciplinary Unit (UED), with 432 vacancies for the custody of provisional detainees and convicts in closed regime and of inmates subjected to differential disciplinary system; and (8) Lemos Brito Prison (PLB), with 771 vacancies, intended for holding inmates sentenced to a closed regime from the capital and countryside counties. Considering their specificities, we categorized the units into (I) COP + CMP + GEOP; (II) CPF; (III) convict units: PLB + UED; and (IV) provisional units: CP + PS.

The prison complex employed 571 AP, according to a nominal personnel list provided by the human resources department of each unit after the consent of directors, the union and the Seap Superintendency. The target population consisted of workers on medical leave (17); on premium leave (15); in process of retirement (6); rotating works, out of service and not employed into a specific unit or sector (37); workers transferred to jobs at Seap or at the union (15); and those in deviation of function (4) – all considered ineligible for data collection. We considered 477 individuals eligible for this study; of these, 46 refused to participate and 30 were not found (the eight units are distributed over a vast area and, often, the AP were performing external activities during the collection period). In the end, 401 (84.1%) individuals participated in the study.

As a research tool, we used a self-reported questionnaire concerning information on sociodemographic characteristics, work aspects and mental health conditions. We approached the AP individually during their shift and requested they fill out the self-reported questionnaire, while remaining available to clarify any doubts about the answers.

The variables used in this study were categorized into three groups: sociodemographic, work and mental health. The sociodemographic characteristics comprised: gender (male or female), age group (23 to 38; 39 to 54; or ≥ 55 years), skin color (white or non-white), education (tertiary or primary/secondary/technical education), marital status (with or without partner) and having children (yes or no).

The work aspects were: role (security, co-ordination/management or administration), working prison unit (I, II, III, IV), work time in years (up to 5; 6 to 20; or ≥ 21 years), institutional training for the position (yes or no), compatibility between position and role (yes or no), having experienced rebellion, escape and/or threats (yes or no), having met inmates outside work (yes or no), officer’s perception on the influence of the environment or work conditions in their health (mental and physical), having contact with inmates most of the time (yes or no) and number of activities that generate emotional stress (changing shifts, opening and closing jail cells, visits, searches, counting inmates, “baculejo” – personal inspection of inmates –, managing internal and external displacement of inmates and conflict situations with inmates, categorized into: none; 1 to 2; 3 to 5; or ≥ 6 activities).

Finally, from mental health conditions came the outcome variable of the study: diagnostic screening of MDD, obtained from the Patient Health Questionnaire-9 (PHQ-9)^8^. This questionnaire is often used in non-psychiatric settings to identify depressive symptoms and perform diagnostic screening for major depression (MD)^9,10^. It consists of nine questions that assess the presence of these symptoms: depressive mood, anhedonia (little interest or pleasure in doing things), sleeping problems, fatigue or lack of energy, change in appetite or weight, feeling guilty or worthless, concentration problems, feeling sluggish or restless and suicidal thoughts. The frequency of each symptom is assessed, based on the last two weeks, on a Likert scale of 0 to 3 with responses ranging from “not at all” (0), “several days” (1), “more than half the days” (2) and “nearly every day” (3)^10^. PHQ-9 was originally developed to identify depressive disorders in primary health care^9^ and allows us to classify its results according to two methods: the simple scoring method, using a cut-off point, and the algorithm method.

This study used the two PHQ-9 for MDD evaluation measures. For the simple score method, we adopted the cut-off point ≥ 10 symptoms, obtained by adding the responses to the instrument. For the algorithm method, we considered the screening positive when five or more symptoms were frequently present (more than half the days); the condition was attested when items related to depressive mood or loss of interest were reported as more than half the days. The item “suicidal ideation” was considered in the sum of the symptoms at any frequency^9,10^.

A recent meta-analysis conducted with 40 studies, comprising 26,902 individuals, of which 14.3% had MDD, presented the following indicators of sensitivity and specificity for this disease, respectively, using the linear method: 56.8% (95%CI 41.2–71.8) and 93.3% (95%CI 87.5–97.3)^14^.

A review with 29 studies on MDD, in turn, estimated that at the cut-off point ≥ 10, PHQ-9 would have sensitivity of 88% (95%CI 83-92) and specificity of 85% (95%CI 82-88)^15^. The simple score method presents higher sensitivity and specificity values than the algorithm method^11^, being widely used to evaluate this outcome^7,8,12,16,17^.

Our exploratory predictive model examined the MDD diagnostic screening by classifying its results according to both methods. The independent variables considered were the AP’s sociodemographic characteristics (gender, age group, skin color, education, marital status and having children) and work aspects (role, work unit, work time, institutional training, compatibility between position and role, having experienced rebellion, escape and/or threat from factions, having met inmates outside of work, having contact with inmate, perception of work influence on one’s own health and number of activities that generate emotional stress). Initially, we selected the independent variables of the study according to the theoretical framework on the topic. The descriptive analysis calculated absolute and relative frequencies; the bivariate analysis calculated the prevalence ratio (PR_crude_) of each variable with the studied outcome. Subsequently, we used the theoretical criterion to place the variables in the multivariate model, considering their importance in the consulted literature. Cox multivariate regression was performed, calculating the adjusted prevalence ratio (PR_adj_) for each independent variable. We inserted the variables in two blocks: sociodemographic characteristics and work aspects, in that order. Variables with PR_adj_ ≥ 1.30 remained in the final model – value that, although arbitrary, has been previously used to identify factors associated with the prevalence of common mental disorders in a census study with medical students^18^. According to Rothman^19^, epidemiological research that measures associations should focus, preferably, on the estimation of effects, rather than the result of statistical inference tests.

Since this is a census study, the use of statistical inference (with statistical tests and presentation of confidence intervals) is not justified, as it applies to studies with probabilistic samples or randomization^19^. Data statistical analysis was performed using SPSS version 22 and Stata 8.0 programs.

This study was approved by the Research Ethics Committee of the School of Medicine of Bahia (FMB) of the Federal University of Bahia (UFBA), under opinion no. 2,464,066 and amendment no. 2,824,557.

## RESULTS

The MDD prevalence in the AP’ diagnostic screening, using the cut-off point ≥ 10 method and algorithm method, was 18.8% (75/400) and 9.3% (37/398), respectively.

In the bivariate analysis, MDD prevalence with cut-off point ≥ 10 was strongly associated (PR_crude_ ≥ 1.30) with the following sociodemographic characteristics: women (PR = 2.29), age groups 23 to 38 and 39 to 54 years, white (PR = 1.32) and tertiary education (PR = 1.71). In the screening by the algorithm method, MDD prevalence was strongly associated with the female gender (PR = 2.61), age group 39 to 54 years (PR = 1.36) and tertiary education (PR = 2.11) (Table 1).

**Table 1:** Prevalence (p, in %) and prevalence ratios (PR) of major depressive disorder (MDD), using two Patient Health Questionnaire-9 (PHQ-9) classification methods, according to sociodemographic characteristics in detention officers (Salvador, Brazil, 2018).

Characteristic	Periodicity		MDD (cut-off point ≥ 10)			MDD (algorithm)		
	n	%	n	p	PR	n	p	PR
Gender								
Female	69	17.2	24	34.8	2.26	13	18.8	2.61
Male	332	82.8	51	15.4	1	24	7.2	1
Age group (years)								
23–38	126	31.5	27	21.4	1.50	12	9.5	1.25
39–54	155	38.8	30	19.4	1.36	16	10.3	1.36
≥ 55	119	29.7	17	14.3	1	9	7.6	1
Skin color								
White	30	7.5	5	16.7	1.32	3	10.0	1.09
Non-white	371	92.5	47	12.7	1	34	9.2	1
Education level								
Tertiary education	254	63.3	56	22.0	1.71	29	11.4	2.11
Primary/secondary/technical education	147	36.7	19	12.9	1	08	5.4	1
Marital status								
Without partner	129	32.2	23	17.8	1	14	10.9	1.28
With partner	272	67.8	52	19.1	1.07	23	8.5	1
Has children								
No	111	27.8	21	18.9	1.03	11	10.0	1.11
Yes	289	72.3	53	18.3	1	26	9.0	1

APs’ work aspects strongly associated (PR_crude_ ≥ 1.30) with MDD prevalence assessed by the cut-off point ≥ 10 method were: role (administrative and security), work time (6 to 20 years), activities incompatible with the position, report that the environment and working conditions interfered with physical health, report that the environment and working conditions interfered with mental health, not have experienced an inmates escape, have suffered threats from factions, perform stress-generating activities and work in the CPF. The MDD prevalence evaluated by the algorithm method was strongly associated with the same characteristics mentioned above, excepting work time, but including not having institutional training for the position (Table 2).

**Table 2:** Prevalence (p, in %) and prevalence ratios (PR) of major depressive disorder (MDD), using two Patient Health Questionnaire-9 (PHQ-9) classification methods, according to characteristics of detention officers’ work (Salvador, Brazil, 2018).

Characteristic	Periodicity		MDD (cut-off point ≥ 10)			MDD (algorithm)		
	n	%	n	p	PR	n	p	PR
Role								
Administrative	22	5.6	7	31.8	2.22	4	18.2	2.98
Security	319	81.8	61	19.1	1.34	30	9.4	1.54
Coordination/management	49	12.6	7	14.3	1	3	6.1	1
Work time (years)								
Up to 5	162	40.4	30	18.5	1.25	15	9.3	1.09
6 to 20	97	24.2	24	24.7	1.67	10	10.3	1.21
≥ 21	142	35.4	21	14.8	1	12	8.5	1
Contact with inmates								
Yes	325	81.0	61	18.8	1.02	30	9.2	1.00
No	76	19.0	14	18.4	1	7	9.2	1
Activities compatible with position								
No	14	3.5	4	28.6	1.55	2	14.3	1.59
Yes	387	96.5	71	18.3	1	35	9.0	1
Institutional training for the position								
No	137	34.2	29	21.2	1.22	16	11.7	1.46
Yes	264	65.8	46	17.4	1	21	8.0	1
Work interferes in physical health								
Yes	356	89.2	72	20.2	4.36	36	10.1	4.39
No	43	10.8	2	4.65	1	1	2.3	1
Work interferes in mental health								
Yes	359	89.8	72	20.1	2.77	34	9.5	1.30
No	41	10.2	3	7.3	1	3	7.3	1
Experienced rebellion								
No	182	45.4	40	22.0	1.38	19	10.4	1.27
Yes	219	54.6	35	16.0	1	18	8.2	1
Experienced scape								
Yes	212	52.9	32	15.1	1	17	8.0	1
No	189	47.1	43	22.8	1.51	20	10.6	1.32
Suffered threat from criminal factions								
Yes	122	30.4	31	25.4	1.62	15	12.3	1.56
No	279	69.6	44	15.8	1	22	7.9	1
Met an inmate outside the prison								
Yes	349	87.0	67	19.2	1.25	33	9.5	1.23
No	52	13.0	8	15.4	1	4	7.7	1
Stress-generating activities								
None	8	2.0	1	12.5	1	1	12.5	1.69
1 to 2 activities	108	26.9	15	13.9	1.12	8	7.4	1
3 to 5 activities	159	39.7	30	18.9	1.51	16	10.1	1.36
6 or more activities	126	31.4	29	23.0	1.84	12	9.5	1.28
Prison unit								
Female unit	36	9.0	11	30.6	1.94	7	19.4	2.52
Convicts units	133	33.2	26	19.6	1.24	12	9.0	1.17
Provisional units	180	44.9	30	16.7	1.06	14	7.8	1.01
Specialized units	52	12.9	8	15.4	1	4	7.7	1

In the final multivariate model, MDD prevalence assessed by the cut-off point ≥ 10 method remained high (PR_adj_ ≥ 1.30) in female AP (PR_adj_ = 2.77), among those who reported that the environment and working conditions interfered with their physical health (PR_adj_ = 3.51), those who were threatened by factions (PR_adj_ = 2.05) and those who reported not having had institutional training for the position (PR_adj_ = 1.38). Emotional stress-generating activities showed an increasing gradient according to their number (none; 1 to 2; 3 to 5; or ≥ 6), with PR_adj_ of 1.00, 1.49, 1.60 and 1.77, respectively. MDD prevalence according to the algorithm method remained high in female AP (PR_adj_ = 3.45), with tertiary education (PR_adj_ = 1.71), who reported that the environment and working conditions interfered with their physical health (PR_adj_ = 6.33), suffered threat from factions (PR_adj_ = 2.14), whose work required frequent contact with inmates (PR_adj_ = 1.48) and who lacked institutional training (PR_adj_ = 1.50) (Table 3).

**Table 3:** Crude (PRcrude) and adjusted (PRadj) prevalence rates of the Cox regression model for factors associated with major depressive disorder (MDD), using two Patient Health Questionnaire-9 (PHQ-9) classification methods, in detention officers in Salvador, Bahia (2018).

Associated factors	PR_crude_	PR_adj_
MDD (cut-off point ≥ 10)		
Gender		
Female	2.29	2.77
Male	1	1
Stress-generating activities		
None	1	1
1 to 2 activities	1.12	1.49
3 to 5 activities	1.52	1.60
6 or more activities	1.84	1.77
Work interferes in physical health		
Yes	4.37	3.51
No	1	1
Suffered threat from criminal factions		
Yes	1.62	2.05
No	1	1
Institutional training for the position		
No	1.22	1.38
Yes	1	1
MDD (algorithm)		
Gender		
Female	2.64	3.45
Male	1	1
Education level		
Tertiary education	2.13	1.75
Primary/secondary/technical education	1	1
Work interferes in physical health		
Yes	4.37	6.33
No	1	1
Suffered threat from criminal factions		
Yes	1.56	2.14
No	1	1
Contact with inmates		
Yes	1.00	1.48
No	1	1
Institutional training		
No	1.47	1.50
Yes	1	1

## DISCUSSION

MDD prevalence in the investigated AP, of 18.8% by cut-off point ≥ 10 and 9.3% by algorithm method, was higher than that reported for the world population (4.4%)^7^. Brazilian Social Security data show that depression is the most reported mood disorder, accounting for almost 40% of all leaves due to common mental disorders and 82% of mood disorders reported in granting benefits with leave^22^.

Studies with AP confirm the finding of higher prevalence of depression in women^4,6,23,24^. Prison work, historically characterized as harsh, violent and hostile, reinforces the greater pressure experienced by women^25,26^. Coexistence with other women in social and family vulnerability and double shifts also maximize exposure to suffering^26^. Of the various factors linked to mental illness in women prison workers in France, however, depressive symptomatology was greatly related to subjective experiences of working conditions, labor social relations and factors outside the prison environment than to objectively measured working conditions^27^.

In our study, AP who reported that the environment and working conditions interfered in their physical health had a MDD prevalence 3.51 times higher, by the cut-off method, and 6.33 times higher, by the algorithm method, than those who did not report this influence. In this context, the presence of physical problems may result from two conditions of high demand at work: physical, by direct musculoskeletal action or other body systems besides the musculoskeletal; and/or mental, when individuals have difficulty in expressing or dealing with their emotions, with these manifestations being somatized in the body^24^. The work activities performed by AP present strong mental demand, which can impact their physical health.

In a 1999 study conducted with the APs of this same Prison Complex^4^, the following factors were associated with the periodicity of complaints of general health complaints: infrastructure conditions of the environment, such as furniture suitability, luminosity, ventilation, availability of materials and quality of housing; work psychosocial aspects, such as satisfaction in performing activities, independence in performing tasks, interpersonal relationships with colleagues and management, and power of organization and hierarchy; and work organization, i.e., repetitiveness of tasks, noise, humidity, team size and pressure from management.

In AP’s work, structural conditions of the environment, equipment without adequate maintenance, low staffing, prison overcrowding and permanent alertness result in high daily demands for performing their tasks^25,28^. Among these demands are the already mentioned tension-enhancing activities, such as changing shifts, opening and closing jail cells, visits, searches, counting inmates, “baculejo,” managing internal and external displacement of inmates and conflict situations with detainees. In our study, the whole of these demands characterized a work of high physical and mental demand, with increasing dose-response gradient: the higher the number of stress-generating activities, the higher the MDD prevalence in AP.

Having suffered threat from factions was a work psychosocial aspect that was associated with the higher MDD prevalence in both methods employed. According to journalistic investigations, the Mata Escura Prison Complex has a link between inmates and organized crime factions that dispute the control of drug trafficking in Bahia. In 2016, the three largest factions totaled 4,053 inmates, including provisional and convicts^29^. The impact of prison work goes beyond the internal context, since the fear and exposure experienced outside prison walls – of meeting inmates and/or their families, for example, or even of living near them – are conditions that can favor stress and affect the mental health of these workers. Threats can also involve AP’s family members, culminating in their mental suffering^30^.

These AP activities involve exposure to inadequate working conditions and environment, which can lead to their suffering and illness^3^. Threats, contact with inmates, tensions, rebellions and escapes are part of the AP’s work context. The risks of proximity to individuals deprived of their liberty include inmate attacks, physical and verbal assaults, lawsuits for escaping prisoners, inquiries, and direct and indirect threats^28,30^. The particularities of prison camp work require that the role be performed by professionals with a stable link, opportunity for permanent training that enables them to perform the role, and structural conditions that ensure their work comfort, physical and psychological, to minimize the risk sickness^28,31^.

A study with AP from the same prison complex revealed a higher level of stress in professionals who reported not having had institutional training for their work in prison, the stress factor being considered relevant for the occurrence of minor psychic disorders in this population^4^. In our study, AP who reported not having had training for the position had a higher MDD prevalence in both methods. Such findings reinforce the importance of institutional actions of continued training for performing prison work. These actions cannot do without understanding the reality of daily work and identifying the main difficulties faced by AP, so that they are not limited to prescriptions of rules and norms, but are institutional actions that build the viability of effective solutions and alternatives to improve life at work.

Recruitment for the position of AP occurs via public tender and has as minimum educational requirement the average education. Meanwhile, the presence of prison workers with tertiary education is an expanding reality^31^. In our study, most AP reported having tertiary education, being those with higher MDD prevalence. Working in prison camps involves threat of loss of stability as a public servant, difficulty of entering the job market and the idea that the AP role is temporary. Perhaps the AP with tertiary education were better equipped to recognize and feel the harmful effects of work on their mental health.

We must also consider the limitations of this study. Due to their cross-sectional nature, the results obtained offer little evidence of causality. Another important limitation was not having investigated aspects of the institution’s organizational dimension and psychosocial aspects of AP’s work. Despite its epidemiological scope, which basically uses quantitative methods, the inclusion of open questions and interviews could have enriched the study and given clues on the subjective aspects experienced by AP.

Comparing the findings of this study may have been limited by the different evaluation instruments for depressive disorders used in the scientific literature, as well as the different cut-off points adopted to analyze the PHQ-9 results. However, MDD screening from both PHQ-9 measurements confirms personal and work context factors associated with depressive disorders, with different prevalences. The study reinforces the use of PHQ-9 and the need for discussions on the different measurements produced by this instrument for understanding depressive disorders.

It was not possible to evaluate the AP on leave, being considered in this research only those who were on duty during data collection, which may have resulted in the *healthy worker’s effect*.

On the other hand, it is worth noting that this was a census study conducted in the largest prison complex in the state of Bahia, with a reasonably high proportion of respondent workers (84.1%). Its census character, including AP of all prison units, allowed to characterize the variability of occupational exposure situations.

## CONCLUSION

This study found a high prevalence of MDD in AP, associated with several characteristics: female gender, contact with inmates, number of stress-generating work activities, lack of training for the position, suffering threat from criminal factions, and reporting that the environment and working conditions interfered in one’s physical health. The results of this research help to define the profile of AP’s professional category and contribute to give visibility to a profession hardly visible socially. These results can be useful to promote strategic actions that minimize the illness and physical and mental suffering of these workers.
