# Supplementary material for: Major depressive disorder in detention officers
Source: Rev Saude Publica. 2021 Apr 5;55:11. doi: 10.11606/s1518-8787.2021055002507 (PMC8023319; doi:10.11606/s1518-8787.2021055002507)
Supplement: Translation

# Translation: Transtorno depressivo maior em agentes penitenciários

**Authors:** Sheila Nascimento Santos, Kionna Oliveira Bernardes Santos, Fernando Martins Carvalho, Rita de Cássia Pereira Fernandes

**Affiliations:** I Brasil Universidade Federal da Bahia. Programa de Pós-Graduação em Saúde, Ambiente e Trabalho. Departamento de Medicina Preventiva e Social. Salvador, Bahia, Brasil

**Keywords:** Prisões, Transtorno Depressivo Maior, Questionário de Saúde do Paciente, Saúde do Trabalhador

## Abstract

**OBJETIVO:**

Identificar fatores associados a transtorno depressivo maior (TDM) em agentes penitenciários.

**MÉTODOS:**

Este estudo de corte transversal incluiu todos os agentes penitenciários do maior complexo prisional do estado da Bahia (Brasil). Num questionário autoaplicado, coletaram-se informações sociodemográficas, ocupacionais e de saúde. A variável de desfecho – TDM – foi avaliada pelo *Patient Health Questionnaire-9* (PHQ-9) e classificada pelo método do ponto de corte ≥ 10 e pelo método de algoritmo. A razão de prevalência (RP) foi a medida de associação utilizada. Conforme regressão multivariada de Cox, as variáveis foram inseridas em dois blocos: características sociodemográficas e aspectos do trabalho, nessa ordem. Apenas as variáveis com RP ajustada (RP_aj_) ≥ 1,30 foram selecionadas para compor os modelos finais.

**RESULTADOS:**

Nos 401 agentes investigados, a prevalência de TDM pelo ponto de corte ≥ 10 (simples) e pelo método de algoritmo foi de 18,8% e 9,3%, respectivamente. A prevalência de TDM pelo ponto de corte ≥ 10 foi maior em agentes do sexo feminino (RP_aj_ = 2,77), que sofreram ameaça de facções (RP_aj_ = 2,05), que não referiram treinamento institucional para o cargo (RP_aj_ = 1,38), que afirmaram que o ambiente e as condições de trabalho interferiam na sua saúde física (RP_aj_ = 3,51) e que exerciam atividades geradoras de tensão (RP_aj_ em gradiente crescente). A prevalência de TDM pelo método de algoritmo foi mais elevada em agentes do sexo feminino (RP_aj_ = 3,45), com escolaridade superior (RP_aj_ = 1,71), que afirmaram que o ambiente e as condições de trabalho interferiam na sua saúde física (RP_aj_ = 6,33), que sofreram ameaça de facções (RP_aj_ = 2,14), que não referiram treinamento institucional (RP_aj_ = 1,50) e que têm contato frequente com internos no trabalho (RP_aj_ = 1,48).

**CONCLUSÃO:**

A alta prevalência de TDM nesses agentes penitenciários associou-se a aspectos sociodemográficos e, principalmente, a aspectos do seu trabalho.

## INTRODUÇÃO

O trabalho no cárcere é uma ocupação estressante, arriscada e estigmatizada pela sociedade^1,2^. O agente penitenciário (AP) é o responsável pela segurança interna dos presídios e dos indivíduos privados de liberdade^3^. Entre outras atribuições, esses profissionais são encarregados, diária e continuamente, por revistar presos, celas e visitantes, abrir e fechar celas prisionais, realizar vigilância interna das unidades, conduzir presos interna e externamente ao presídio e disciplinar suas refeições^4^.

O trabalho dos AP caracteriza-se pelo contato direto com detentos, regulamentações de serviço específicas, ambientes com infraestrutura precária, profissão sem reconhecimento legal enquanto função de segurança pública, pressão, sobrecarga, desvalorização e falta de reconhecimento social da importância de seu ofício. Os AP estão expostos a altos riscos psicossociais decorrentes de seu trabalho, o que os torna propensos a transtornos mentais comuns, transtornos psicóticos, dependência química, alcoolismo, estresse, ansiedade, *burnout*, afastamento do trabalho e síndromes depressivas^3,5,6^.

Os transtornos depressivos destacam-se entre os transtornos mentais pela alta prevalência na população, associados a sofrimento ou incapacidade, que podem afetar atividades sociais e profissionais^7^. Nesse sentido, este trabalho objetivou identificar fatores associados a transtorno depressivo maior (TDM) em AP.

## MÉTODOS

Foi realizado um estudo epidemiológico de corte transversal. A população foi constituída por todos os AP, sem restrição de sexo ou idade, pertencentes ao quadro efetivo da Secretaria de Administração Penitenciária e Ressocialização (Seap) da Bahia, distribuídos entre cargos de chefia, administração e segurança e lotados no Complexo Penitenciário da Mata Escura, na Região Metropolitana de Salvador (Bahia, Brasil). A coleta de dados foi realizada de agosto a dezembro de 2018.

O Complexo Penitenciário tem oito unidades que apresentam características próprias relacionadas à condição jurídica do preso e à natureza de cada atividade ali exercida: (1) Centro de Observação Penal (COP), com 96 vagas, onde são realizados exames gerais e criminológicos de presos condenados, bem como de presos de recolhimento especial, provisórios ou condenados, maiores de 60 anos de idade, e outros exames autorizados pela Corregedoria-Geral de Justiça; (2) Central Médica Penitenciária (CMP), com 16 vagas, que fornece atendimento médico emergencial a presos de ambos os sexos, das diversas Unidades Prisionais do estado da Bahia; (3) Grupo Especial de Operações Prisionais (GEOP), destinado exclusivamente à lotação de agentes de intervenção especializada de inteligência prisional; (4) Conjunto Penal Feminino (CPF), com 132 vagas, destinado à custódia de presas condenadas em regimes fechado e semiaberto de comarcas do interior do estado e de presas provisórias da Comarca de Salvador, sendo quase todos os AP dessa unidade do sexo feminino; (5) Cadeia Pública de Salvador (CP), com 1.092 vagas, que custodia presos provisórios da comarca da capital e, excepcionalmente, do interior do estado, quando autorizada pela Corregedoria-Geral de Justiça; (6) Presídio de Salvador (PS), com 784 vagas, que abriga presos provisórios, semelhantes aos encontrados na CP; (7) Unidade Especial Disciplinar (UED), com 432 vagas destinadas à custódia de presos provisórios e condenados em regime fechado e de internos submetidos a regime disciplinar diferenciado; e (8) Penitenciária Lemos Brito (PLB), com 771 vagas, que se destina ao recolhimento de presos condenados a regime fechado das comarcas da capital e do interior do estado. Considerando suas especificidades, as unidades foram categorizadas em: (I) COP + CMP + GEOP; (II) CPF; (III) unidades de condenados: PLB + UED; e (IV) unidades de provisórios: CP + PS.

O complexo prisional empregava 571 AP, segundo lista nominal de pessoal fornecida pelo setor de recursos humanos de cada unidade após consentimento de diretores, do sindicato e da Superintendência da Seap. A população-alvo continha trabalhadores em licença médica (17); em licença-prêmio (15); em processo de aposentadoria (6); trabalhadores rotativos, fora do serviço e que não estavam lotados em unidade ou setor específico (37); trabalhadores transferidos para postos de trabalho como os da Seap ou do sindicato (15); e os que se encontravam em desvio de função (4) – todos considerados não elegíveis para a coleta de dados. Foram considerados elegíveis para este estudo 477 indivíduos; destes, 46 se recusaram a participar e 30 não foram encontrados (as oito unidades se distribuem numa imensa área e, muitas vezes, os AP estavam cumprindo atividades extramuros durante o período de coleta). Ao final, 401 (84,1%) indivíduos participaram do estudo.

Como instrumento de pesquisa, foi utilizado um questionário autoaplicável contendo informações sobre características sociodemográficas, características de trabalho e condições de saúde mental. Os pesquisadores abordavam individualmente o AP durante o seu período de trabalho e solicitavam que preenchesse o questionário autoaplicável, enquanto permaneciam disponíveis para esclarecimento de quaisquer dúvidas de entendimento do texto.

As variáveis utilizadas neste estudo foram categorizadas em três grupos: sociodemográficas, de trabalho e de saúde mental. As características sociodemográficas são: sexo (masculino ou feminino), faixa etária (23 a 38; 39 a 54; ou ≥ 55 anos), cor da pele (branca ou não branca), escolaridade (ensino superior ou fundamental/médio/técnico), situação conjugal (com ou sem companheiro) e ter filhos (sim ou não).

Já as características de trabalho são: função (segurança, coordenação/diretoria ou administrativa), unidade prisional de trabalho (I, II, III ou IV), tempo de trabalho em anos (até 5; 6 a 20; ou ≥ 21 anos), treinamento institucional para o cargo (sim ou não), compatibilidade entre cargo e função exercida (sim ou não), ter vivenciado rebelião, fuga e/ou ameaças (sim ou não), ter encontrado presos fora do trabalho (sim ou não), percepção do agente sobre a interferência do ambiente e das condições de trabalho na sua saúde (física e mental), ter contato com internos na maior parte do tempo (sim ou não) e número de atividades geradoras de tensão emocional (rendição, abrir e fechar cadeia, visita, revista, contagem de internos, “baculejo” – inspeção pessoal de internos –, conduzir deslocamento interno e externo de detento e situação de conflito com preso, categorizadas em: nenhuma; 1 a 2; 3 a 5; ou ≥ 6 atividades).

Por fim, das condições de saúde mental proveio a variável de desfecho do estudo: triagem diagnóstica de TDM, obtida a partir do *Patient Health Questionnaire-9* (PHQ-9)^8^. Esse questionário é frequentemente utilizado em ambientes não psiquiátricos para identificar sintomas depressivos e realizar triagem diagnóstica de depressão maior (DPM)^9,10^. Ele contém nove questões que avaliam a presença desses sintomas, que consistem em: humor deprimido, anedonia (perda de interesse ou prazer em fazer as coisas), problemas com o sono, cansaço ou falta de energia, mudança no apetite ou peso, sentimento de culpa ou inutilidade, problemas de concentração, sentir-se lento ou inquieto e pensamentos suicidas. A frequência de cada sintoma é avaliada, com base nas últimas duas semanas, em uma escala Likert de 0 a 3 com respostas que variam entre “nenhuma vez” (0), “poucos dias” (1), “mais da metade dos dias” (2) e “quase todos os dias” (3)^10^. O PHQ-9 foi originalmente desenvolvido para identificar transtornos depressivos na atenção primária à saúde^9^ e permite classificar seus resultados segundo dois métodos: o método de pontuação simples, usando um ponto de corte, e o método de algoritmo.

Este estudo utilizou as duas medidas de avaliação do PHQ-9 para TDM. Para o método da pontuação simples, foi adotado o ponto de corte ≥ 10 sintomas, obtido pela soma das respostas ao instrumento. Para o método de algoritmo, a triagem foi considerada positiva quando cinco ou mais sintomas estiveram presentes com frequência (mais da metade dos dias); além disso, a condição foi atestada quando os itens relativos ao humor deprimido ou à perda de interesse foram relatados com a frequência de mais da metade dos dias. O item referente à ideação suicida foi considerado no somatório dos sintomas em qualquer uma das frequências de apresentação^9,10^.

Uma recente metanálise realizada com 40 estudos, abarcando 26.902 indivíduos, dos quais 14,3% tinham DPM, apresentou os seguintes indicadores de sensibilidade e especificidade para essa doença, respectivamente, usando o método linear: 56,8% (IC95% 41,2–71,8) e 93,3% (IC95% 87,5–97,3)^14^.

Já uma revisão com 29 estudos sobre DPM estimou que, com o ponto de corte ≥ 10, o PHQ-9 teria sensibilidade de 88% (IC95% 83–92) e especificidade de 85% (IC95% 82–88)^15^. O método da pontuação simples apresenta maiores valores de sensibilidade e especificidade que o método de algoritmo^11^, sendo amplamente utilizado na avaliação desse desfecho^7,8,12,16,17^.

O modelo preditivo exploratório deste estudo investigou a triagem diagnóstica da TDM classificando seus resultados segundo os dois métodos. As variáveis independentes consideradas foram as características sociodemográficas (sexo, faixa etária, cor da pele, escolaridade, situação conjugal e ter filhos) e do trabalho (função, unidade de trabalho, tempo de trabalho, treinamento institucional, compatibilidade entre cargo e função exercida, ter vivenciado rebelião, fuga e/ou ameaça de facções, ter encontrado detento fora do trabalho, ter contato com detento, percepção do efeito do trabalho sobre a própria saúde e número de atividades geradoras de tensão emocional) dos AP. Inicialmente, a seleção das variáveis independentes do estudo foi realizada de acordo com o referencial teórico para o tema. Na análise descritiva calcularam-se frequências absolutas e relativas. A análise bivariada calculou a razão de prevalência (RP_bruta_) de cada variável com o desfecho estudado. Posteriormente, o critério teórico foi utilizado para inserir as variáveis no modelo multivariado, considerando a importância destas na literatura consultada. Foi realizada regressão multivariada de Cox, com cálculo da razão de prevalência ajustada (RP_aj_) para cada variável independente. As variáveis foram inseridas em dois blocos: características sociodemográficas e aspectos do trabalho, nesta ordem. Permaneceram no modelo final as variáveis que apresentaram RP_aj_ ≥ 1,30 – valor que, apesar de arbitrário, foi anteriormente utilizado para identificar fatores associados à prevalência de transtornos mentais comuns em um estudo censitário com estudantes de medicina^18^. Segundo Rothman^19^, a pesquisa epidemiológica que mede associações deve focar, preferencialmente, a estimativa dos efeitos, e não o resultado de testes de inferência estatística.

Considerando ser este um estudo censitário, não se justifica o uso da inferência estatística (com testes estatísticos e apresentação de intervalos de confiança), uma vez que esta se aplica a estudos com amostras probabilísticas ou randomização^19^. A análise estatística dos dados foi feita com o auxílio dos programas SPSS versão 22 e Stata 8.0.

Este estudo foi aprovado pelo Comitê de Ética em Pesquisa da Faculdade de Medicina da Bahia (FMB) da Universidade Federal da Bahia (UFBA), com parecer nº 2.464.066 e emenda nº 2.824.557.

## RESULTADOS

A prevalência de TDM na triagem diagnóstica dos AP, usando o método de ponto de corte ≥ 10 e o método de algoritmo, foi de 18,8% (75/400) e 9,3% (37/398), respectivamente.

Na análise bivariada, a prevalência de TDM com o ponto de corte ≥ 10 associou-se fortemente (RP_bruta_≥ 1,30) com as seguintes características sociodemográficas: sexo feminino (RP = 2,29), faixas etárias de 23 a 38 e de 39 a 54 anos, cor da pele branca (RP = 1,32) e escolaridade superior (RP = 1,71). Já na triagem pelo método de algoritmo, a prevalência de TDM associou-se fortemente com o sexo feminino (RP = 2,61), a faixa etária de 39 a 54 anos (RP = 1,36) e a escolaridade superior (RP = 2,11), conforme exposto na Tabela 1.

**Tabela 1:** Prevalência (p, em %) e razões de prevalência (RP) de transtorno depressivo maior (TDM), usando dois métodos de classificação do *Patient Health Questionnaire-9* (PHQ-9), segundo características sociodemográficas em agentes penitenciários (Salvador, Brasil, 2018).

Característica	Frequência		TDM (ponto de corte ≥ 10)			TDM (algoritmo)		
	n	%	n	p	RP	n	p	RP
Sexo								
Feminino	69	17,2	24	34,8	2,26	13	18,8	2,61
Masculino	332	82,8	51	15,4	1	24	7,2	1
Faixa etária (anos)								
23–38	126	31,5	27	21,4	1,50	12	9,5	1,25
39–54	155	38,8	30	19,4	1,36	16	10,3	1,36
≥ 55	119	29,7	17	14,3	1	9	7,6	1
Cor da pele								
Branca	30	7,5	5	16,7	1,32	3	10,0	1,09
Não branca	371	92,5	47	12,7	1	34	9,2	1
Escolaridade								
Ensino superior	254	63,3	56	22,0	1,71	29	11,4	2,11
Ensino fundamental/médio/técnico	147	36,7	19	12,9	1	08	5,4	1
Situação conjugal								
Sem companheiro	129	32,2	23	17,8	1	14	10,9	1,28
Com companheiro	272	67,8	52	19,1	1,07	23	8,5	1
Tem filhos								
Não	111	27,8	21	18,9	1,03	11	10,0	1,11
Sim	289	72,3	53	18,3	1	26	9,0	1

As características do trabalho dos AP fortemente associadas (RP_bruta_ ≥ 1,30) à prevalência de TDM avaliada pelo método do ponto de corte ≥ 10 foram: função (administrativa e segurança), tempo de trabalho (de 6 a 20 anos), atividades não compatíveis com o cargo, referir que o ambiente e as condições de trabalho interferiam na saúde física, referir que o ambiente e as condições de trabalho interferiam na saúde mental, não ter presenciado fuga de detentos, ter sofrido ameaça de facções, realizar atividades geradoras de tensão e trabalhar no CPF. A prevalência de TDM avaliada pelo método de algoritmo associou-se fortemente às mesmas características citadas para o ponto de corte, exceto pelo tempo de trabalho, porém incluindo não ter treinamento institucional para o cargo (Tabela 2).

**Tabela 2:** Prevalência (p, em %) e razões de prevalência (RP) de transtorno depressivo maior (TDM), usando dois métodos de classificação do *Patient Health Questionnaire-9* (PHQ-9), segundo características do trabalho de agentes penitenciários (Salvador, Brasil, 2018).

Característica	Frequência		TDM (ponto de corte ≥ 10)			TDM (algoritmo)		
	n	%	n	p	RP	n	p	RP
Função								
Administrativa	22	5,6	7	31,8	2,22	4	18,2	2,98
Segurança	319	81,8	61	19,1	1,34	30	9,4	1,54
Coordenação/diretoria	49	12,6	7	14,3	1	3	6,1	1
Tempo de trabalho (anos)								
Até 5	162	40,4	30	18,5	1,25	15	9,3	1,09
6 a 20	97	24,2	24	24,7	1,67	10	10,3	1,21
≥ 21	142	35,4	21	14,8	1	12	8,5	1
Contato com internos								
Sim	325	81,0	61	18,8	1,02	30	9,2	1,00
Não	76	19,0	14	18,4	1	7	9,2	1
Atividades compatíveis com cargo								
Não	14	3,5	4	28,6	1,55	2	14,3	1,59
Sim	387	96,5	71	18,3	1	35	9,0	1
Treinamento institucional para o cargo								
Não	137	34,2	29	21,2	1,22	16	11,7	1,46
Sim	264	65,8	46	17,4	1	21	8,0	1
Trabalho interfere na saúde física								
Sim	356	89,2	72	20,2	4,36	36	10,1	4,39
Não	43	10,8	2	4,65	1	1	2,3	1
Trabalho interfere na saúde mental								
Sim	359	89,8	72	20,1	2,77	34	9,5	1,30
Não	41	10,2	3	7,3	1	3	7,3	1
Presenciou rebelião								
Não	182	45,4	40	22,0	1,38	19	10,4	1,27
Sim	219	54,6	35	16,0	1	18	8,2	1
Presenciou fuga								
Sim	212	52,9	32	15,1	1	17	8,0	1
Não	189	47,1	43	22,8	1,51	20	10,6	1,32
Sofreu ameaça de facções								
Sim	122	30,4	31	25,4	1,62	15	12,3	1,56
Não	279	69,6	44	15,8	1	22	7,9	1
Encontrou detento fora da cadeia								
Sim	349	87,0	67	19,2	1,25	33	9,5	1,23
Não	52	13,0	8	15,4	1	4	7,7	1
Atividades geradoras de tensão (n)								
Nenhuma	8	2,0	1	12,5	1	1	12,5	1,69
1 a 2 atividades	108	26,9	15	13,9	1,12	8	7,4	1
3 a 5 atividades	159	39,7	30	18,9	1,51	16	10,1	1,36
6 ou mais atividades	126	31,4	29	23,0	1,84	12	9,5	1,28
Unidade prisional								
Unidade feminina	36	9,0	11	30,6	1,94	7	19,4	2,52
Unidades de condenados	133	33,2	26	19,6	1,24	12	9,0	1,17
Unidades de provisórios	180	44,9	30	16,7	1,06	14	7,8	1,01
Unidades especializadas	52	12,9	8	15,4	1	4	7,7	1

No modelo multivariado final, a prevalência de TDM avaliada pelo método do ponto de corte ≥ 10 permaneceu elevada (RP_aj_ ≥ 1,30) em AP do sexo feminino (RP_aj_ = 2,77), entre os que referiram que o ambiente e as condições de trabalho interferiam na sua saúde física (RP_aj_ = 3,51), entre os que foram ameaçados por facções (RP_aj_ = 2,05) e entre os que referiram não ter tido treinamento institucional para o cargo (RP_aj_ = 1,38). As atividades geradoras de tensão emocional apresentaram gradiente crescente de acordo com o número dessas atividades (nenhuma; 1 a 2; 3 a 5; ou ≥ 6), com RP_aj_ de 1,00, 1,49, 1,60 e 1,77, respectivamente. A prevalência de TDM segundo o método de algoritmo permaneceu elevada em AP do sexo feminino (RP_aj_ = 3,45), com escolaridade superior (RP_aj_ = 1,71), que referiram que o ambiente e as condições de trabalho interferiam na sua saúde física (RP_aj_ = 6,33), que sofreram ameaça de facções (RP_aj_ = 2,14), cujo trabalho exigia contato frequente com internos (RP_aj_ = 1,48) e que não tiveram treinamento institucional (RP_aj_ = 1,50), como mostra a Tabela 3.

**Tabela 3:** Razões de prevalência bruta (RPbruta) e ajustada (RPaj) do modelo de regressão de Cox para fatores associados a transtorno depressivo maior (TDM), usando dois métodos de classificação do *Patient Health Questionnaire-9* (PHQ-9), em agentes penitenciários de Salvador, Bahia (2018).

Fatores associados	RP_bruta_	RP_aj_
TDM (ponto de corte ≥ 10)		
Sexo		
Feminino	2,29	2,77
Masculino	1	1
Atividades geradoras de tensão		
Nenhuma	1	1
1 a 2 atividades	1,12	1,49
3 a 5 atividades	1,52	1,60
6 ou mais atividades	1,84	1,77
Trabalho interfere na saúde física		
Sim	4,37	3,51
Não	1	1
Sofreu ameaça de facções		
Sim	1,62	2,05
Não	1	1
Treinamento institucional para o cargo		
Não	1,22	1,38
Sim	1	1
TDM (algoritmo)		
Sexo		
Feminino	2,64	3,45
Masculino	1	1
Escolaridade		
Ensino superior	2,13	1,75
Ensino fundamental, médio ou técnico	1	1
Trabalho interfere na saúde física		
Sim	4,37	6,33
Não	1	1
Sofreu ameaça de facções		
Sim	1,56	2,14
Não	1	1
Tem contato com internos		
Sim	1,00	1,48
Não	1	1
Treinamento institucional		
Não	1,47	1,50
Sim	1	1

## DISCUSSÃO

A prevalência de TDM nos AP investigados, de 18,8% pelo ponto de corte ≥ 10 e de 9,3% pelo método do algoritmo, foi mais alta do que a relatada para a população mundial (4,4%)^7^. Dados da Previdência Social brasileira mostram que a depressão é o transtorno de humor mais notificado, representando quase 40% de todos os afastamentos por transtornos mentais comuns e 82% dos transtornos de humor notificados na concessão de benefícios com afastamento^22^.

Estudos com AP confirmam o achado de maior prevalência de depressão no sexo feminino^4,6,23,24^. O trabalho penitenciário, historicamente caracterizado como rude, violento e hostil, reforça a maior pressão vivenciada pelas mulheres^25,26^. A convivência com outras mulheres em vulnerabilidade social e familiar e a dupla jornada de trabalho também potencializam a exposição ao sofrimento^26^. Entretanto, dos diversos fatores ligados ao adoecimento mental em mulheres trabalhadoras do cárcere na França, a sintomatologia depressiva esteve mais relacionada a experiências subjetivas das condições de trabalho, relações sociais laborais e fatores fora do ambiente prisional do que às condições laborais objetivamente mensuradas^27^.

Neste estudo, AP que referiram que o ambiente e as condições de trabalho interferiam na sua saúde física apresentaram prevalência de TDM 3,51 vezes maior, pelo método do ponto de corte, e 6,33 vezes maior, pelo método de algoritmo, do que aqueles que não referiram essa influência. Nesse contexto, a existência de problemas físicos pode decorrer de duas condições de alta exigência no trabalho: a física, por ação osteomuscular direta ou de outros sistemas corporais além do musculoesquelético; e/ou mental, quando o indivíduo tem dificuldade em expressar ou lidar com suas emoções, sendo essas manifestações somatizadas no corpo^24^. As atividades de trabalho exercidas pelo AP apresentam forte demanda mental, o que pode impactar sua saúde física.

Em estudo realizado em 1999 com AP desse mesmo Complexo Penitenciário^4^, os seguintes fatores se associaram à frequência de queixas gerais de saúde: condições infraestruturais do ambiente, tais como adequação de mobiliário, luminosidade, ventilação, disponibilidade de materiais e qualidade do alojamento; aspectos psicossociais do trabalho, como satisfação no desempenho das atividades, independência na realização de tarefas, relações interpessoais com colegas e chefia, e poder de organização e hierarquia; e organização do trabalho, ou seja, repetitividade de tarefas, barulho, umidade, tamanho da equipe e pressão da direção.

No trabalho dos AP, condições estruturais do ambiente, equipamentos sem manutenção adequada, baixo efetivo de pessoal, superlotação prisional e o estado de alerta permanente iresultam em altas exigências cotidianas para o desenvolvimento de suas tarefas^25,28^. Entre essas exigências estão as já mencionadas atividades potencializadoras de tensão, como rendição, abertura e fechamento da cadeia, visitas, revista, contagem de internos, “baculejo”, condução do deslocamento interno e externo de detentos e situações conflitantes com os presos. Neste estudo, o conjunto dessas demandas caracterizou um trabalho de alta exigência física e mental, com gradiente dose-resposta crescente: quanto maior o número de atividades geradoras de tensão, maior a prevalência de TDM nos AP.

Neste estudo, ter sofrido ameaça de facções foi um aspecto psicossocial do trabalho que se associou a maior prevalência de TDM nos dois métodos empregados. Segundo investigações jornalísticas, no Complexo Penitenciário da Mata Escura há vinculação entre detentos e facções do crime organizado que disputam o controle do tráfico de drogas na Bahia. As três maiores facções somavam, em 2016, 4.053 internos, entre presos provisórios e condenados^29^. O impacto do trabalho no cárcere vai além do contexto interno, pois o medo e a exposição vivenciada fora dos muros prisionais – de encontrar presos e/ou seus familiares, por exemplo, ou até mesmo de residir próximo a eles – são condições que podem favorecer o estresse e afetar a saúde mental desses trabalhadores. As ameaças podem envolver também os familiares dos AP, culminando no seu sofrimento mental^30^.

As atividades dos AP estudados envolvem exposição a condições e ambiente de trabalho inadequados, que podem levar ao sofrimento e ao adoecimento desses trabalhadores^3^. Ameaças, contato com internos, tensões, rebeliões e fugas fazem parte do contexto laboral do AP. Os riscos da proximidade com indivíduos privados de liberdade incluem ataques de detentos, agressões físicas e verbais, ações judiciais por fuga dos presos, sindicâncias e ameaças diretas e indiretas^28,30^. As particularidades do trabalho no campo prisional requerem que a função seja desempenhada por profissionais com vínculo estável, oportunidade de capacitação permanente, que os habilite ao exercício da função, e condições estruturais que assegurem seu conforto laboral, físico e psíquico, de modo a minimizar o risco de adoecerem^28,31^.

Estudo com AP do mesmo complexo prisional revelou maior nível de estresse em profissionais que referiram não ter tido treinamento institucional para seu trabalho no cárcere, sendo o fator estresse considerado relevante para a ocorrência de distúrbio psíquico menor nessa população^4^. No presente estudo, os AP que referiram não ter tido treinamento para o cargo apresentaram maior prevalência de TDM, em ambos os métodos. Tais achados reforçam a importância das ações institucionais de capacitação continuada para o exercício da função no cárcere. Essas ações não podem prescindir do conhecimento da realidade do trabalho cotidiano e da identificação das principais dificuldades enfrentadas pelos AP, a fim de que não se limitem a prescrições de regras e normas, mas sejam ações institucionais que construam a viabilidade de soluções e alternativas efetivas para melhorar a vida no trabalho.

A seleção para o cargo de AP ocorre via concurso público e tem como exigência mínima de escolaridade a formação média. Entretanto, a presença de trabalhadores do cárcere com formação superior é uma realidade em expansão^31^. Neste estudo, a maioria dos AP referiu ter escolaridade superior, sendo esses os que apresentaram prevalência mais elevada de DPM. O trabalho no campo prisional envolve a ameaça de perda da estabilidade de servidor público, a dificuldade de inserção no mercado de trabalho e a ideação de que a função de AP é temporária. Talvez os AP com escolaridade superior estivessem mais habilitados para reconhecer e sentir os efeitos nocivos do trabalho sobre a sua saúde mental.

É importante considerar as limitações deste estudo. Devido ao seu caráter transversal, os resultados obtidos oferecem poucas evidências de causalidade. Outra limitação importante foi não ter investigado aspectos da dimensão organizacional da instituição e aspectos psicossociais do trabalho dos AP. Apesar de seu escopo epidemiológico, que basicamente usa métodos quantitativos, a inclusão de questões abertas e entrevistas poderia ter enriquecido o trabalho e dado pistas sobre os aspectos subjetivos vivenciados pelos AP.

A comparação dos achados deste estudo pode ter sido limitada devido aos diferentes instrumentos de avaliação para transtornos depressivos utilizados na literatura científica, bem como pelos diferentes pontos de corte adotados para analisar os resultados do PHQ-9. Entretanto, o rastreio de DPM a partir de ambas as medições do PHQ-9 confirma fatores pessoais e do contexto de trabalho associados aos transtornos depressivos, com diferentes prevalências. O estudo reforça a utilização do PHQ-9 e a necessidade de discussões sobre as diferentes mensurações produzidas por esse instrumento para compreensão dos transtornos depressivos.

Não foi possível avaliar os AP afastados da função, sendo considerados nesta pesquisa apenas os que estavam exercendo a profissão quando da coleta de dados, o que pode ter resultado no efeito do trabalhador sadio.

Por outro lado, vale salientar que este foi um estudo censitário realizado no maior complexo prisional do estado da Bahia, com proporção de trabalhadores respondentes razoavelmente alta (84,1%). O caráter censitário do estudo, incluindo AP de todas as unidades prisionais, permitiu caracterizar a variabilidade de situações de exposição ocupacional.

## CONCLUSÃO

Este estudo encontrou alta prevalência de TDM em AP, associada a várias características: sexo feminino, contato com internos, número de atividades laborais geradoras de tensão, falta de treinamento para o cargo, sofrer ameaça de facções criminosas e referir que o ambiente e as condições de trabalho interferiam na saúde física. Os resultados desta pesquisa ajudam a definir o perfil da categoria profissional dos AP e contribuem para dar visibilidade a uma ocupação pouco visível socialmente. Esses resultados podem ser úteis para promover ações estratégicas que minimizem o adoecimento e o sofrimento físico e mental desses trabalhadores.
